# Prognostication in Different Heart Failure Phenotypes: The Role of Circulating Biomarkers

**DOI:** 10.5772/62797

**Published:** 2016-03-16

**Authors:** Alexander E. Berezin

**Affiliations:** 1 State Medical University, Zaporozhye, Ukraine

**Keywords:** Heart Failure Phenotypes, Biomarkers, Prognostication, Risk Stratification

## Abstract

Heart failure (HF) is multifactorial syndrome with high cardiovascular (CV) morbidity and mortality rates associated with an increasing prevalence worldwide. Measuring plasma levels of circulating biomarkers, i.e., natriuretic peptides, cardiac-specific troponins, metabolomic intermediates, Galectin-3, ST2, cardiotrophin-1, soluble endoglin and growth differentiation factor 15, may assist in the prognostication of HF development. However, the role of biomarker models in the prediction of an early stage of HF with a preserved ejection fraction (HFpEF) and HF with a reduced ejection fraction (HFrEF) is not still understood. This review explores the knowledge regarding the utility of cardiac biomarkers, aiming to reclassify patients with different phenotypes of HF. The review reports that several biomarkers reflected on subsequently alter collagen turnover, cardiac fibrosis and inflammation, which might have diagnostic and predictive value in HFpEF and HFrEF. The best candidates for determining the early stage of HF development were sST2, Galectin-3, CT-1 and GDF-15. However, increased plasma concentrations of these biomarkers were not specific to a distinct disease group of HFpEF and HFrEF. Finally, more investigations are required to determine the role of novel biomarkers in the prediction of HF and the determination of the early stages of HFpEF and HFrEF development.

## 1. Introduction

Heart failure (HF) remains an important clinical entity that has increased in prevalence worldwide due to improved survival after a HF diagnosis [1, 2]. Recent studies have shown sufficient differences in the aetiology, pathophysiology, clinical presentation and outcomes, as well as the prognosis, between HF with a preserved ejection fraction (HFpEF) and HF with a reduced ejection fraction (HFrEF) [3–5].

HFpEF is a phenotypic and heterogeneous clinical syndrome characterized by cardiovascular (CV) disease and dysmetabolic and inflammatory states associated with both advanced age and various non-CV co-morbidities, which finally lead to the impairment of myocardial structure and function, unless under the condition of declining global EF <45% [6, 7]. Although global left ventricular EF >50% is currently used to differentiate between reduced and preserved cardiac pump function, this cut-off point is widely discussed as a likely inadequate criterion [8, 9] However, old age, being female, suffering from diabetes mellitus, hypertension, atrial fibrillation and chronic kidney disease are strong predictors of HFpEF's development [10–12]. Based on evidence from endomyocardial biopsies, some of the specific cardiac structural phenotypes to be targeted in HFpEF may be represented by myocyte hypertrophy and interstitial fibrosis [13, 14]. HFrEF has been described as a disease of aged elderly subjects, with a male predominance that is frequently associated with dilation cardiomyopathy, ischaemia, inflammatory and diabetic aetiology, and rarely with arterial and pulmonary hypertension [15, 16]. Cell loss due to ischaemia, apoptosis and necrosis, myocardial inflammation associated with oxidative stress, expanded interstitial fibrosis leading to the disintegrity of the cardiac wall, increased passive myocardial stiffness, the worsening of cardiac configuration and contractile function are common in HFrEF's development [17].

Many questions remain unanswered regarding differences in the molecular signals that initiate the development of HFpEF and HFrEF [18]. In this context, it might be possible to appropriately stratify at risk HFpEF and HFrEF patients by using biomarkers. Recently, brain natriuretic peptides, cardiac specific troponins, metabolomic intermediates, Galectin-3, ST2, cardiotrophin-1, soluble endoglin, growth differentiation factor 15 and other new biological markers associated with HF's development have been widely investigated [5, 6, 12, 13, 17, 19]. However, the current data on the interrelationship of these biomarkers and phenotypes of HF are limited. The aim of the review is devoted to the accumulation of knowledge regarding the utility of cardiac biomarkers, aiming to reclassify patients with different phenotypes of HF.

## 2. Biomarkers in HF Risk Stratification

The routine use of biomarkers might help to stratify the patients with HFrEF and HFpEF at higher risk of death and clinical outcomes. The current guidelines — the 2012 European Society of Cardiology (ESC) Guidelines for the Diagnosis and Treatment of Acute and Chronic Heart Failure and the 2013 American College of Cardiology Foundation/American Heart Association (ACCF/AHA) Guideline for the Management of Heart Failure — are well accepted by many clinicians regarding HFrEF's prognostication. Indeed, HFpEF is the one that really requires the improvement of biomarkers for diagnosis and prognosis. In this context, many biological markers, which reflect several faces of the pathogenesis of HF, have been investigated in detail, but only natriuretic peptides, soluble ST2, Galectin-3 and highly sensitive cardiac-specific troponins have been validated thus far. Table 1 offers summarized evidence regarding the predictive role of biomarkers in patients with different HF phenotypes.

## 3. Brain Natriuretic Peptides

Within the last two decades, cardiac natriuretic peptides (BNP and NT-proBNP) have been defined as biomarkers that we may use to screen for LV systolic dysfunction in patients with symptoms suggestive of HF. BNP and NT-proBNP are now included in the current guidelines for HF diagnosis, management and risk assessment because of their high specificity and sensitivity [19]. Despite BNP and NT-proBNP improving discrimination modestly for HF above and beyond conventional risk factors, and substantially improving the risk classification for HF, peak concentrations of BNP and NT-proBNP and serial measurements of NT-proBNP levels in longitude are not able to allow the differentiation of HF phenotypes [20, 21]. However, there were important differences in the prognostic value of NT-proBNP in HFpEF versus HFrEF in the NT- proBNP-guided arm of the TIME-CHF study [22]. NT-proBNP has demonstrated less prognostic value in HFpEF as compared to HFrEF, and has not predicted a development of HFpEF or HFrEF. NT-proBNP lost significance as a risk stratifier in ambulatory patients with stable HF and probably also in those who have HFpEF. There are attampts to use of sing sample measurement of mid-regional atrial natriuretic peptide (mr-ANP) and NT-proANP in order to screen HFpEF and HFrEF in individuals, when the diagnosis of HF is not obvious. In this setting, the diagnostic value and prognostic ability for HF-related mortality and CV hospitalization for both mr-ANP and NT-proANP were not superior to those of NT-proBNP [23].

## 4. Cardiac Troponins

Recent studies have shown that elevated levels of highly sensitive cardiac troponin I (hs-cTnI) and T (hs-cTnT) as biomarkers of subclinical myocardial injury may provide to be clinically useful prognostic information, concerning both the future risk of HF's manifestation in asymptomatic subjects and the risk of fatal events and primary/re-admissions in the hospital in those with already established symptomatic acute, acutely decompensated and chronic stable HF related to ischaemic and non-ischaemic causes [24–27]. Moreover, cardiac troponin mutations are considered a cause of impaired relaxation in the mutant cardiac myocytes due to myofibril hypersensitivity to Ca^2+^ [28].

**Table 1.** Predictive role of biomarkers in patients with different HF phenotypes

Biomarkers	Patient population	The most important findings	References
Natriuretic peptides	Exerted dyspnoea	Predictor of HF risk manifestation, risk of admission in the hospital and HF-related deaths	[19]
	Known HFpEF and HFrEF	Biomarkers independently predicted HF-related outcomes, CV mortality, all-cause death, admission in the hospital, but they did not predict a development of HFpEF or HFrEF	[19, 20, 21]
Cardiac troponins	Ischaemia-induced HF	Predictors of HF manifestation risk in asymptomatic subjects Predictors of death in HFpEF and HFrEF	[24, 25]
	Ischaemia-induced and non-ischaemia-related HF	Predictors of death, primary/re-admissions in the hospital	[25–27]
Galectin-3	General population	Prognosticator of HF risk, risk of death from any cause	[33]
	Known HF patients	Predictor of CV death, HF-related deaths, primary and re-admission in the hospital	[35–37]
Soluble ST2	General population	Predictor of higher risk of all-cause mortality, HF manifestation	[42]
	Known HF patients	Independent predictor of CV deaths, HF-related deaths, admission in the hospital	[44]
Cardiotrophin-1	Known ischaemia-induced HF patients	Predictor of CV clinical outcomes	[53, 54]
Endoglin	Patients at higher risk of CV disease	Predictor of CV events/ outcomes, HF manifestation	[58–60]
Growth differentiation factor 15	Patients with known CV disease	Predictor of HF manifestation	[64, 65]
	Known HF patients	Predictor of HF-related outcomes	[68]

Abbreviation: CV, cardiovascular; HF, heart failure; HFpEF, HF with preserved ejection fraction; HFrEF, HF with reduced ejection fraction.

Cardiac-specific troponins exhibited the strongest associations with hospitalization, survival and outcomes in cases of HF; there are expectations regarding the ability of troponins to emerge as an aetiology-dependent relation to phenotypes of HF. Seliger et al. [29] hypothesized that hs-cTnT would identify HF risk among older adults with left ventricular hypertrophy (LVH). In the Cardiovascular Health Study, its authors found that the adjusted risk of HFrEF was 7.8 times higher among those with the highest tertile of hs-cTnT and LVH (HR=7.83; 95% CI: 4.43–13.83). Patients with LVH and longitudinal increases in hs-cTnT or NT-proBNP were approximately three times more likely to develop HF (primarily HFrEF), compared with those without LVH and with stable biomarkers. Thus, in this study, the authors were not able to find sufficient advantages regarding hs-cTnT compared NT-proBNP in order to characterize sub-phenotypes of HF. In another study, Neeland et al. [30] reported that identifying a malignant sub-phenotype of LVH was the better predictive surrogate marker than a limited elevated level of hs-cTnT, and even increased NT-proBNP among asymptomatic individuals with a high risk of progression to HF and CV death in the general population. Therefore, there was evidence that the higher levels of cTnT and NT-proBNP correlated well with the risk of HF in older adults, but were not associated with phenotypes of HF [31]. Overall, the circulating level of the cell injury biomarker is not a powerful tool for HF-phenotype detection.

## 5. Systematic Metabolomic Biomarkers

Zordoky et al. [32] suggested that a systematic metabolomic analysis would reveal a novel metabolomic fingerprint of HFpEF that will help us to understand its pathophysiology and assist us in establishing new biomarkers for its diagnosis. Compared to non-HF control, HFpEF patients demonstrated higher serum concentrations of acylcarnitines, carnitine, creatinine, betaine and amino acids, and lower levels of phosphatidylcholines, lysophosphatidylcholines and sphingomyelins. Medium- and long-chain acylcarnitines and ketone bodies were higher in HFpEF than in HFrEF patients. The authors suggested that this abovementioned metabolomic fingerprint has been utilized to identify two novel panels of metabolites that can separate HFpEF patients from both non-HF controls and HFrEF patients. However, this assumption requires further investigation.

### 5.1 Galectin-3

It has been suggested that various alternative biomarkers might offer insight into the different pathways of HF's pathophysiology, and that they might help to identify individuals in the general population at higher risk of developing HF and patients with known chronic HF with poor outcomes [33]. Galectin-3 is a soluble beta galactoside-binding lectin produced by activated macrophages that bind and activate the fibroblasts [34]. Currently, Galectin-3 is considered a biomarker that mediates an important link between inflammation and fibrosis, which plays a pivotal role in CV remodelling. The pathogenetic role of Galectin-3 in the various settings of pressure overload, neuroendocrine activation, hypertension, coronary artery disease/myocardial infarction, atrial fibrillation and HF has been established.

Galectin-3 has emerged as a predictive value for the onset of HF in apparently healthy patients and has been found to be a surrogate marker of a worse prognosis, mortality and re-admission in HF [35, 36]. However, serial measurements of Galectin-3 levels in ambulatory HF patients might not be of benefit [37].

In the context of determining the different phenotypes of HF, the measurement of circulating Galectin-3 might have a significant value because elevated levels of Galectin-3 were found in patients with impaired LV diastolic function, but without symptomatic HF [38]. Gurel et al. [39] reported that Galectin-3 could be a promising biomarker for the detection of LV diastolic dysfunction in patients undergoing maintenance haemodialysis. It has been suggested that this biomarker could be a useful surrogate for structural and functional abnormality of the myocardium among individuals at higher risk of HFpEF development, especially that associated with hypertension, coronary artery disease and diabetes [40, 41]. However, there is no irrefutable evidence regarding the clinically significant advantages of Galectin-3 in predicting HFpEF's evolution compared with HFrEF's development.

### 5.2 ST2

Soluble ST2 (sST2), a peptide belonging to the interleukin-1 receptor family, is secreted by cardiomyocytes and cardiac fibroblasts under mechanical strain, and is thus regarded as a biomarker of myocardial fibrosis, cardiac stretching and CV remodelling [42, 43]. Measurement of sST2 levels is useful for death risk stratification and prognosis prediction in HF patients, beyond other CV risk factors [44]. The sST2 concentration showed a weak correlation with the NYHA functional class, LFEF, other cardiac performances and renal function [45, 46]. Recent studies have shown that sST2 may have a special superiority as a risk predictor in HFpEF and HFrEF as compared with natriuretic peptides and Galectin-3 [47, 48]. However, there are no current data on the predictive value of sST2 concentrations for HFpEF or HFrEF development.

### 5.3 Cardiotrophin-1

Cardiotrophin-1 (CT-1) is a member of the interleukin 6 cytokine superfamily and one of the endogenous ligands for gp130 signalling pathways in the heart, with controversial biological effects. CT-1 is able to induce hypertrophic growth and contractile dysfunction in cardiomyocytes, as well as having potent hypertrophic and survival effects on cardiac myocytes [49]. CT-1 is closely associated with many CV diseases, i.e., hypertension, myocardial infarction and HF, and exhibits a cardioprotective effect in ischaemia-reperfusion injury during CABG and angioplasty [50]. Recent clinical studies have shown that CT-1 levels are increased in HF patients, and that it is significantly correlated with the LV mass index, suggesting that CT-1 plays an important role in structural LV remodelling [51, 52]. Increased cardiotrophin-1 plasma levels might predict the presence of an inappropriate LV mass merge in hypertensive subjects [53], and the development and progression of HF [54]. Moreover, CT-1 is elevated in patients with HFpEF and is associated with NT-proBNP and estimated LV filling pressures [55]. Whether increased serum CT-1 may provide additional information to aid risk stratification in the development of HFrEF or HFpEF is not completely clear.

### 5.4 Soluble endoglin

Endoglin (also known as CD105) is a membrane co-receptor for transforming growth factor-β, which is released into the circulation in a soluble form and which disrupts TGFβ1 signalling in the endothelium, thereby promoting inflammation, endothelial dysfunction, cardiac fibrosis and vascular remodelling [56, 57]. Endoglin is required for vascular barrier function, endothelial survival and homeostasis of the adult microvasculature, although endoglin is expressed in cardiac fibroblasts and may modulate profibrogenic actions of angiotensin II [58]. A recent clinical study has revealed that the expression of endoglin is increased in patients with atherosclerosis and that the endoglin level is thought to predict CV events in patients with chronic coronary artery disease after PCI [59]. There is evidence regarding the predictive role of elevated serum endoglin in patients with pre-eclampsia [60]. In patients with HFrEF, elevated soluble endoglin levels predicted elevated LV end-diastolic pressures, and correlated well with the New York Heart Association class, irrespective of LVEF, as well as with both atrial and brain natriuretic peptides [56, 61]. The ability of soluble endoglin in prediction of HFpEF and HFrEF is not understood, while there are expectations regarding the role of this biomarker for prognostication in LV dysfunction at early onset. However, extended scrutiny is required to receive more information for testing this assumption.

### 5.5 Growth differentiation factor 15

Growth differentiation factor 15 (GDF-15) is a stress-responsive cytokine, which belongs to the super family of the transforming growth factor beta [62]. GDF-15 is widely presented in the wide spectrum of various cells and plays a pivotal role in inflammation, cell growth and differentiation. Elevated GDF-15 was found in patients with established CV diseases (hypertension, stable coronary artery disease, acute coronary syndrome, myocardial infarction, ischaemic and non-ischaemic-induced cardiomyopathies, HF, atrial fibrillation), type-two diabetes mellitus, chronic renal disease, infection and liver cirrhosis and malignancy [63].

Recent studies have revealed that GDF-15 was associated with the NYHA class, NT-proBNP and exercise capacity, suggesting that the marker has diagnostic and potentially prognostic value in HF [64–66]. It has been suggested that GDF-15 might categorize HFrEF and predict major HF-related clinical outcomes [67]. Chan et al. [68] reported that the plasma levels of GDF15 in HFpEF and HFrEF were similar. Therefore, there was an independent verification of the prognostic utility of GDF15 in HFrEF and HFrEF. The authors have shown that GDF15 was a significant independent predictor for composite outcome, even after adjusting for important clinical predictors including hsTnT and NT-proBNP [68]. Overall, GDF15 was not able to assist in detecting the early stage of HFpEF, and this biomarker has produced very limited evidence regarding the determination of diastolic dysfunction.

## 6. Conclusion

Several reports have shown that biomarkers reflecting the differentiation of fibroblasts into myofibroblasts, subsequently altering collagen turnover, cardiac fibrosis and inflammation, might have diagnostic and predictive value in HFpEF and HFrEF. The biomarkers with most predictive value in determining the early stage of HF's development were sST2, Galectin-3, CT-1 and GDF-15. However, increased plasma concentrations of these biomarkers were not specific for a distinct disease group of HFpEF and HFrEF. Finally, more investigations are required to determine the role of novel biomarkers in predicting HF and the determination of the early stage of HFpEF and HFrEF's development.

## Abbreviations

BNP: – brain natriuretic peptide
CAD: – coronary artery disease
CABG: – coronary artery bypass grafting
GDF: – growth differentiation factor
CT-1: – cardiotrophin-1
CV: – cardiovascular
HF: – heart failure
HFpEF: – heart failure with preserved ejection fraction
HFrEF: – heart failure with reduced ejection fraction LV – left ventricle
PCI: – percutant coronary angioplasty procedure
